# Design, Synthesis, Cytotoxic Evaluation and Molecular Docking of New Fluoroquinazolinones as Potent Anticancer Agents with Dual EGFR Kinase and Tubulin Polymerization Inhibitory Effects

**DOI:** 10.3390/ijms19061731

**Published:** 2018-06-11

**Authors:** Mohamed F. Zayed, Sahar Ahmed, Saleh Ihmaid, Hany E. A. Ahmed, Heba S. Rateb, Sabrin R. M. Ibrahim

**Affiliations:** 1 Pharmacognosy and Pharmaceutical Chemistry Department, College of Pharmacy, Taibah University, Al-Madinah Al-Munawarah 41477, Saudi Arabia; sihmaid@taibahu.edu.sa (S.I.); heahmad@taibahu.edu.sa (H.E.A.A.); hsahmed@taibahu.edu.sa (H.S.R.); sribrahim@taibahu.edu.sa (S.R.M.I.); 2 Pharmaceutical Chemistry Department, Faculty of Pharmacy, Al-Azhar University, Cairo 11884, Egypt; 3 Department of Medicinal Chemistry, Faculty of Pharmacy, Assiut University, Assuit 71526, Egypt; 4 Pharmaceutical Organic Chemistry Department, Faculty of Pharmacy, Al-Azhar University, Cairo 11884, Egypt; 5 Department of Pharmaceutical and Medicinal Chemistry, Pharmacy College, Misr University for Science and Technology, Cairo 12568, Egypt; 6 Department of Pharmacognosy, Faculty of Pharmacy, Assiut University, Assiut 71526, Egypt

**Keywords:** design, fluoroquinazolinone, cytotoxicity, EGFR kinase, tubulin inhibitors

## Abstract

A series of new fluoroquinazolinone **6**–**8** and **10a**–**g** derivatives was designed, prepared and screened for their in vitro cytotoxic activity against human cancer cell lines MCF-7 and MDA-MBA-231. Compounds **6** (IC_50_ = 0.35 ± 0.01 µM), **10f** (IC_50_ = 0.71 ± 0.01 µM), **10d** (IC_50_ = 0.89 ± 0.02 µM) and **10a** (IC_50_ = 0.95 ± 0.01 µM) displayed broad spectrum anticancer activity better than the reference drug gefitinib (IC_50_ = 0.97 ± 0.02 µM) against MCF-7. Compounds **10e** (IC_50_ = 0.28 ± 0.02 µM), **10d** (IC_50_ = 0.38 ± 0.01 µM), **7** (IC_50_ = 0.94 ± 0.07 µM) and **10c** (IC_50_ = 1.09 ± 0.01 µM) showed better activity than the reference gefitinib (IC_50_ = 1.30 ± 0.04 µM) against MDA-MBA-231. Moreover, EGFR and tubulin inhibition assays were performed for the highest active derivatives and showed remarkable results comparing to the reference drugs. In order to assess and explain their binding affinities, molecular docking simulation was studied against EGFR and tubulin binding sites. The results obtained from molecular docking study and those obtained from cytotoxic screening were correlated.

## 1. Introduction

Quinazolines belong to a famous class of heterocyclic compounds displaying a diverse and important range of therapeutic activities [1,2]. They are used as antihypertensive [3], antibacterial [4,5], antiviral [6], anti-inflammatory [7], antidiabetic [8], anticonvulsant, analgesic [1,9] and anticancer medications [10,11,12,13,14,15]. Many quinazolines were reported as anticancer agents having multi-target features [12,13,14]. The targets of action of anticancer quinazolines include inhibition of different enzymes, like epidermal growth factor receptor (EGFR), Aldose reductase (AR), dihydrofolate reductase (DFR), folate thymidylate synthase (FTS), cyclic guanosine monophosphate (cGMP) phosphodiesterase, erythroblastosis oncogene B2 (erB2) tyrosine kinase, and cellular-sarcoma (c-Src) tyrosine kinase. Other quinazolines yield their anticancer activity by inhibition of DNA repairing system or tubulin polymerization. There are many other derivatives have dual EGFR/tubulin polymerization inhibitors, like amide derivatives and quinoxalines [16,17,18,19]. Many efforts have been aimed at finding safe and potent molecules with the present chemotherapeutic agents [10,11,12,13,14,15].

**Figure 1:**
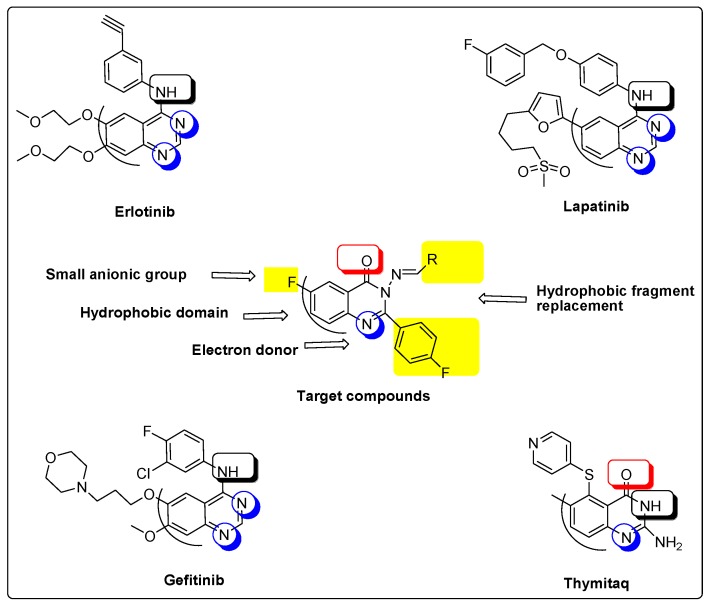
Structural similarities of the reference anticancer quinazolines lapatinib, erlotinib, gefitinib, thymitaq and our designed compounds.

Gefitinib, lapatinib and erlotinib are well-known anticancer drugs with quinazoline nucleus that target epidermal growth factor (EGFR) protein kinase [14,15]. Thymitaq also is a well-known quinazoline anticancer drug that works as a thymidylate synthase inhibitor [12,13]. The modeling study of these drugs, shown in Figure 1, revealed that all of these anticancer models have quinazoline moieties containing different substituents. The quinazoline nucleus contains a hydrophobic domain (aromatic ring system) and two electron donor atoms (2N) merged into a heterocyclic ring. The hydrophobic domain joins to different substituents of the aliphatic or heterocyclic ring with different electronic environment systems, while the heterocyclic ring part is substituted in positions 4 or 2. In our previous studies [12,14], we reported that structural adjustments through halogen substitution of quinazoline nucleus in position 6 and phenyl substitution in position 3 improved anticancer activity. Moreover, Fluorine substitution could improve the overall pharmacokinetics and pharmacodynamics of the molecule by improving solubility, selectivity, bioavailability and metabolic stability [15,16]. In addition, bioisosteric replacement of hydrogen by fluorine in position 4 from the phenyl ring makes electronic modulation to reinforce and enhance the binding interaction process [12,17].

Based on the excellent anticancer activity of quinazolinones and the effective action of fluoride substitution, we performed this study to present new quinazolinones having the following:

1. Two fluoride substitutions. One of them attached directly at position 6 and the other one attached indirectly through 4-fluorophenyl at position 2 of quinazolinone.
2. Different hydrophobic fragments with different electronic environment to enhance hydrophobic interactions, therefore obtaining better binding and better activity. This was done by joining the arylidene moiety at position 3 of quinazolinone. This moiety attached to different substituted aromatic rings. This configuration could target different areas of the EGFR protein kinase binding site to produce more selective molecules. Figure 1 shows the structural similarity of the reference anticancer quinazolines lapatinib, erlotinib, gefitinib, thymitaq and title compounds.

## 2. Results and Discussion

### 2.1. Chemistry

**Scheme 1:**
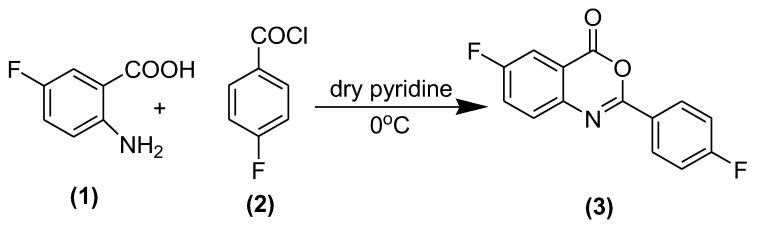
Synthesis of 6-fluoro-2-(4-fluorophenyl)benzoxazinone (**3**).

**Scheme 2:**
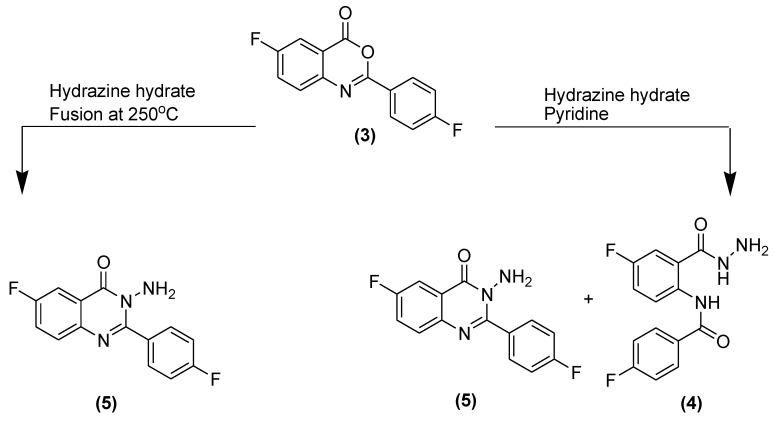
Synthesis of hydrazide derivatives (**4**) and (**5**).

**Scheme 3:**
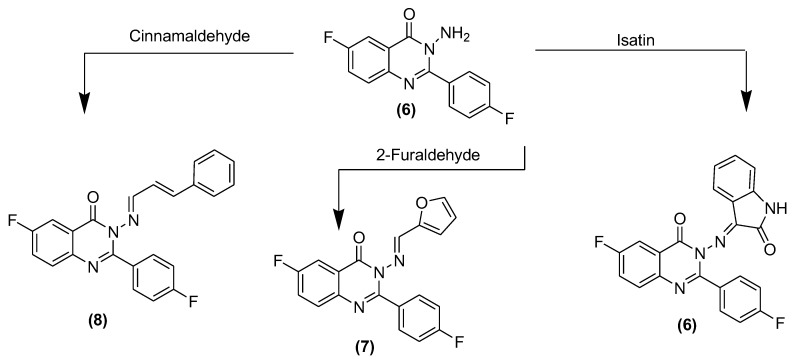
Synthesis of substituted fluoroquinazolinone derivatives (**6**), (**7**) and (**8**).

**Scheme 4:**
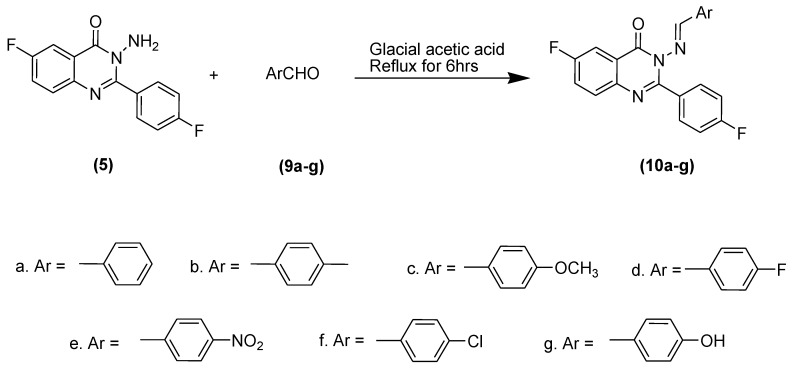
Synthesis of substituted fluoroquinazolinone derivatives (**10a**–**g**).

Preparation of the title compounds of substituted quinazolin-4(3*H*)-one (**6**–**8**) and (**10a**–**g**) is depicted in Scheme 1, Scheme 2, Scheme 3 and Scheme 4. This approach includes three reactions. The first reaction is benzoylation reaction accompanied by ring closure of 2-amino-5-flurobenzoic acid (**1**) by stirring it with 4-flurobenzoylchloride (**2**) to obtain 6-fluoro-2-(4-fluorophenyl)benzoxazinone (**3**).

The second reaction is nucleophilic displacement of the oxygen of substituted benzoxazinone (**3**) by refluxing with hydrazine hydrate in dry pyridine. This reaction afforded a mixture of 2-(4-fluorobenzamido)-5-fluorobenzohydrazide (**4**) and 3-amino-6-fluoro-2-(4-fluorophenyl) quinazolin-4(3*H*)-one (**5**). In order to avoid the ring opening, we performed this reaction by fusion at 250 °C to get the closed form only (**5**).

The third reaction is Schiff’s reaction. It is the nucleohilic addition reaction of aminoquinazolinone (**5**) with different derivatives of aromatic aldehydes or ketones with different electronic environments to obtain 3-(substituted benzylideneamino)-6-fluoro-2-(4-fluorophenyl) quinazoline-4(3*H*)-ones. Indole dione (isatin) was used as an example of aromatic ketone since it has a unique structure containing (C=O) and (NH) incorporated into bulky hydrophobic moiety. These groups could help strong ligand-receptor interaction by forming hydrogen bonding or strong hydrophobic interaction.

### 2.2. Cytotoxicity Screening

The 2,3-disubstituted-6-fluoro-3*H*-quinazolin-4-ones (**6**–**8**) and (**10a**–**g**) were subjected to cytotoxic screening using two types of breast cancer cell lines (MCF-7) and (MDA-MBA-231) using MTT assay [17,18,19]. The IC_50_ values of screening are listed in Table 1.

**Figure 2:**
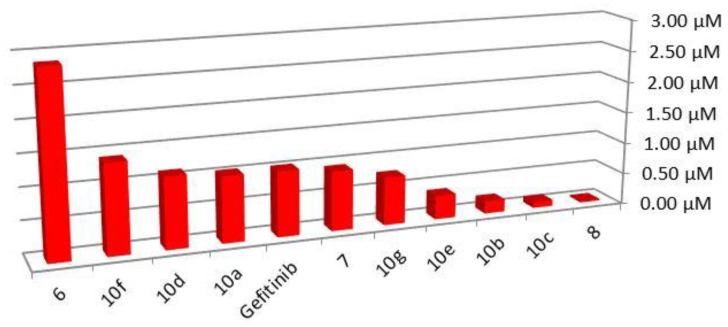
1/IC_50_ values for cytotoxic screening of title compounds against MCF-7 cell line sorted from the least active one (**8**) to the most active one (**6**). Compounds **6**, **10f**, **10d** and **10a** display better activity than the reference gefitinib.

The IC_50_ values using MCF-7 cell line show that all title compounds have significant activity. Compounds **6**, **10f**, **10d** and **10a** display better activity than the reference gefitinib against MCF-7 cell line. Compound **6** (IC_50_ = 0.35 ± 0.01 µM), the most active compound, contains iminoindolin-2-one substitution at position 3 of the quinazolinone nucleus. Iminoindolin-2-one has two hydrogen bonding entities (NH) and (C=O), which could help in the tight binding interaction of this ligand with the receptor site by hydrogen bonding. Moreover, the high lipophilic character of this unit comparing to the other single aromatic units might increase the biological activity. Compound **8** (IC_50_ = 36.57 ± 1.81 µM), the least active compound, contains a 3-phenylallylideneamino unit at position 3 of quinazolinone ring system, and this unit has two double bonds. These bonds increase the electrophilic characters and might negatively affect the biological activity. The other compounds in this series have intermediate activity between these two compounds ranging from IC_50_ = 0.71 ± 0.01 µM to IC_50_ = 10.43 ± 1.14 µM. The order of activity of title compounds can be arranged as **6** > **10f** > **10d** > **10a** > **7** > **10g** >**10e** > **10b** > **10c** > **8**. Figure 2 shows the 1/IC_50_ values for all derivatives using MCF-7 cell line and explains the variations between them compared to the reference gefitinib.

**Figure 3:**
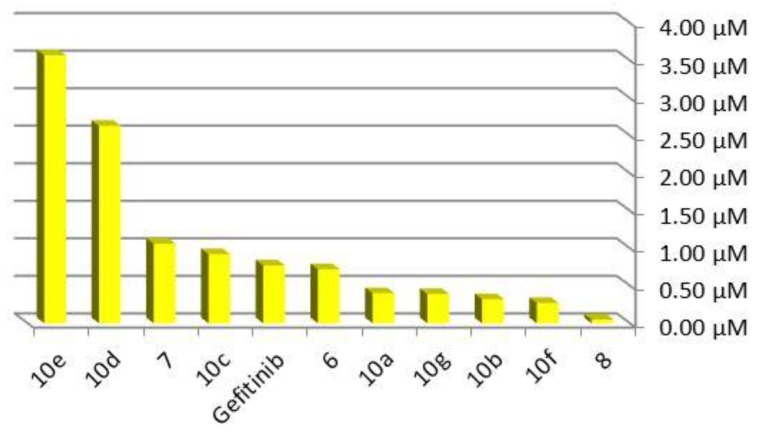
1/IC_50_ values for cytotoxic screening of title compounds against MDA-MBA-231 cell line sorted from the least active one (**8**) to the most active one (**10e**). Compounds **10e**, **10d**, **7** and **10c** display better activity than the reference gefitinib.

The IC_50_ values using MDA-MBA-231 cell line show good activity for all title compounds. Compounds **10e**, **10d**, **7** and **10c** have better activity than gefitinib on the cancer cell line. The most active compound **10e** (IC_50_ = 2.61 ± 0.14 µM) has 4-nitrobenzylideneamino containing a NO_2_ entity, which has the ability to form a hydrogen bond, and this could help improve the binding of the ligand-receptor interaction. Compound **8** (IC_50_ = 21.64 ± 1.4 µM)), the least active compound against this cell line, was also the least active against MCF-7, and this could be explained based on the presence of the allyl moiety, which increases the electrophilicity and could result in unfavorable ligand-receptor interactions. The other compounds show intermediate activity between these two compounds. This activity ranges from IC_50_ = 0.38 ± 0.01 µM and IC_50_ = 3.76 ± 0.22 µM. The cytotoxic action of the title compounds can be arranged as follows: **10e** > **10d** > **7** > **10c** > **6** > **10a** > **10g** > **10b** > **10f** > **8**. Figure 3 shows the 1/IC_50_ values for all derivatives using MDA-MBA-231 cell line and explains the variations between them compared to the reference gefitinib. A further molecular docking study was performed to rationalize these results and explain the means of binding between these derivatives and the receptor site. 

**Figure 4:**
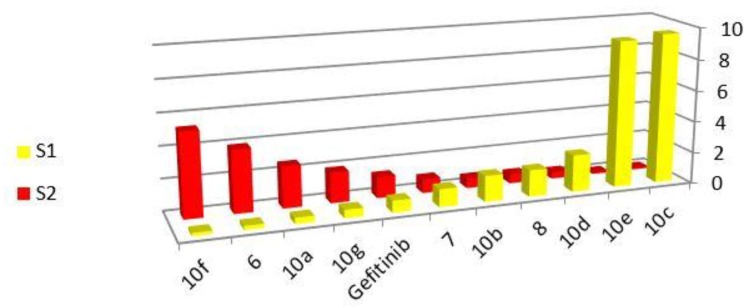
Selectivity of title compounds toward the two cell lines MCF-7 and MDA-MBA-231. Compounds are arranged according to their selectivity order. Increasing yellow color (S1) indicates increasing selectivity to MDA-MBA-231, while increasing red color (S2) indicates increasing selectivity to MCF-7.

Study of selectivity reactivity relationship explained that compounds having methoxy benzylidene amine (**10c**), 4-nitro benzylidene amine (**10e**), 4-fluoro benzylidene amine (**10d**), Phenylallylidene amine (**8**), 4-methyl benzylidene amine (**10b**) and (furan-2-yl)methylene amine (**7**) substitution at the 3rd position of 2,6-disubstituted quinazolinone are more selective to MDA-MBA-231 than MCF-7. On the other hand, compounds having 4-chloro benzylidene amine (**10f**), 3-iminoindolin-2-one (**6**), benzylidene amine (**10a**), and 4-hydroxy benzylidene amine **(10g)** substitution at the 3rd position of 2,6-disubstituted quinazolinone are more selective to MCF-7 than MDA-MBA-231. Table 2 and Figure 4 show selectivity of the compounds according to their orders.

### 2.3. EGFR Assay

It is well known that epidermal growth factor receptors (EGFR) are overexpressed in most tumors [19]. These receptors are very important targets for the action of anticancer agents [13,14]. Many quinazolines, like gefitinib, lapatinib, erlotinib and canertinib, have been reported as strong inhibitors of these receptors, and have great therapeutic potential in cancer treatment [10,11,12,13,14,15]. The previous facts encouraged us to examine the EGFR inhibitory activity of the highest active compounds. The results obtained above showed that compound (**6**) is the most active compound on MCF-7, while compound (**10e**) is the most active one on MDA-MBA-231. These two compounds were screened for their inhibitory activity against EGFR-TK. The two compounds displayed better inhibitory activity than the reference gefitinib. Compound **6**, containing an iminoindolin-2-one substituent at position 3 of the quinazolinone nucleus, had IC_50_ = 75.2 nM on the MCF-7 cell line. Compound **10e**, containing 4-nitrobenzylideneamino at position 3 of the quinazolinone nucleus, had IC_50_ = 170.08 nM on MDA-MBA-231 cell line. The reference gefitinib had IC_50_ = 78.04 nM and 299 nM against the two cell lines, as shown in Table 3. From the previous results, we notice the potency of these derivatives as EGFR inhibitors, but we need further exploration to get more details about their mode of binding with EGFR binding site.

### 2.4. Tubulin Polymerization Inhibition Assay

Microtubules are involved in cellular division and other essential cellular processes. They are generated by polymerization of α and β-tubulin [19,20]. Inhibition of this process leads to stopping cellular mitotic division, and therefore, tubulin polymerization inhibition is an essential target in treating various types of cancer [10,19]. Based on the excellent activity of quinazoline derivatives as tubulin inhibitors [20], and in order to know the effect of these compounds on tubulin polymerization process, a tubulin assay was performed for the active compound (**10d**) because it displayed good activity against the two cell lines together (IC_50_ = 0.89 ± 0.02 µM)/(MCF-7) and (IC_50_ = 0.382114 ± 0.01 µM)/(MDA-MBA-231). The tubulin assay for this compound showed (IC_50_ = 9.25 µM) compared to the reference colchicine (IC_50_ =7.35 µM). This result displays significant activity for this compound as a tubulin polymerization inhibitor, and this might explain the high cytotoxic activity of these new derivatives based on the tubulin polymerization inhibition mechanism. Further rationalizations and details about the mode of binding of this compound with the tubulin binding site compared to the reference colchicine were explained by the molecular docking study.

### 2.5. Molecular Docking Study into the EGFR Binding Site

**Figure 5:**
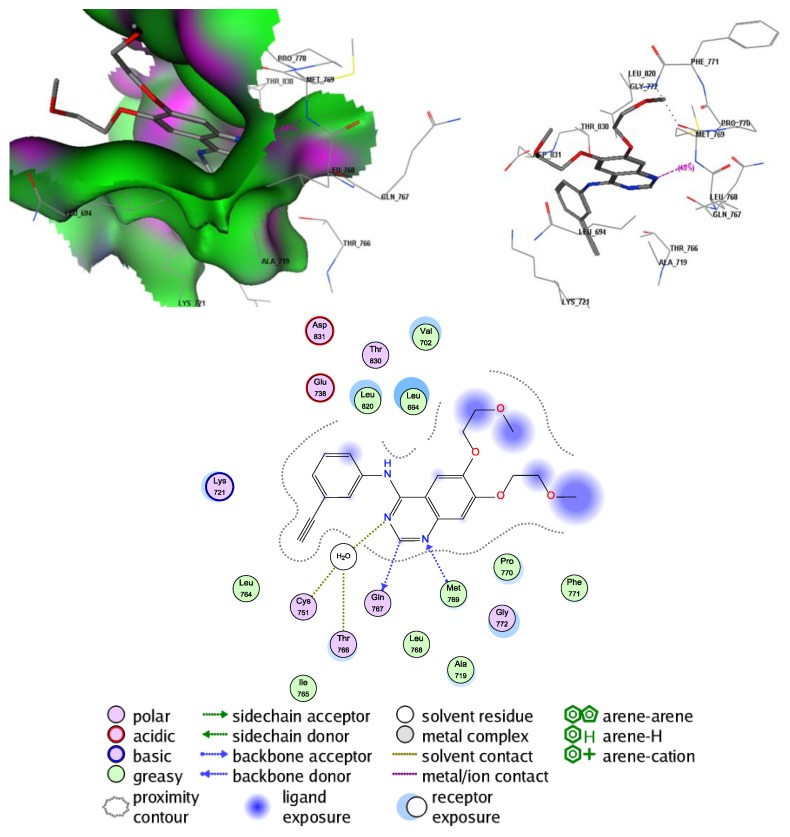
3D and 2D interactions of erlotinib with EGFR binding site show one type of hydrogen bonding.

Molecular docking was performed for the most active compound, **6**, to explain the predicted mode of binding of the title compounds with EGFR and compare binding affinity of title compounds to that of the reference gefitinib. This molecule was docked inside the binding site of erlotinib in the crystalline structure of EGFR (PDB; 1M17) using the AutoDock software. The co-crystallized erlotinib revealed a medium-strength H-bonding interaction (40%) based on the distance between hydrogen acceptor and hydrogen donor. This interaction was between the N1 atom of quinazoline nucleus and OH of *Met769* (distance 2.7 Å). There was also a weak hydrophobic interaction for the aromatic ring and two hydrophobic interactions for the aliphatic side chain (-CH2-O-CH3). Figure 5 shows 2D and 3D interactions of erlotinib with the receptor-site.

**Figure 6:**
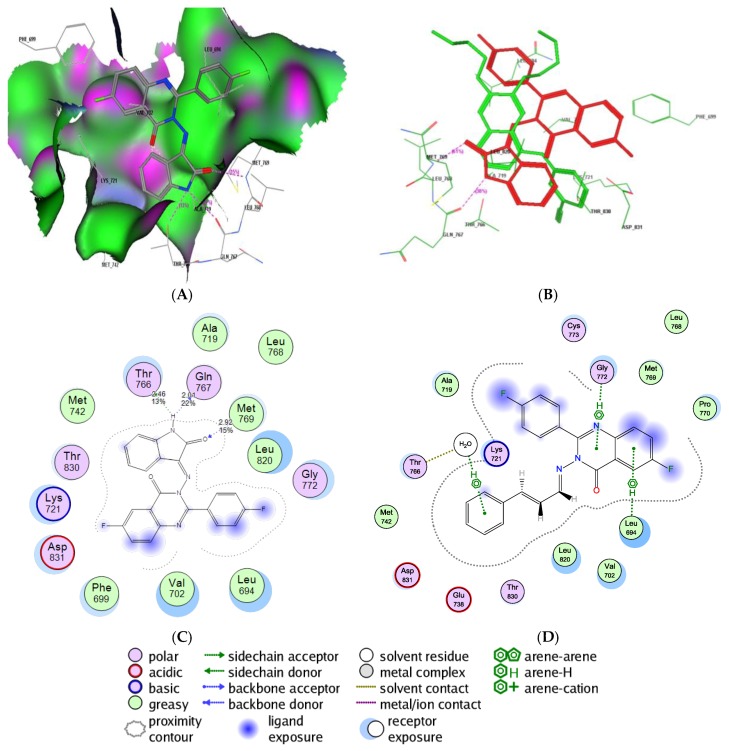
3D and 2D interactions of compounds **6** and **8** with the EGFR binding site compared to erlotinib reference drug. Image (**A**) shows 3D interactions of compound **6** with EGFR binding site, Image (**B**) shows compound 6 superimposed with erlotinib. Image (**C**) shows 2D interactions of compound **6** with EGFR binding site and image (**D**) shows 2D interactions of compound **8** with EGFR binding site.

Compound **6** was able to occupy the EGFR binding site the same way through a hydrogen bonding interaction with *Met769*, in addition to other hydrogen bonds, which led to better binding with the receptor-site than that of erlotinib. Compound **6** comprised 15% hydrogen bonding between (C=O) and (OH) of *Met769* (distance 2.92 Å), 22% hydrogen bonding between (NH) and *Gln767* (distance 2.01 Å) and 13% hydrogen bonding between (NH) and *Thr766 (*distance 2.46 Å). These interactions led to a better binding energy score, −19.53 Kcal/mol for compound **6**, than that of erlotinib, −15.57 Kcal/mol. Figure 6 shows 3D and 2D interactions of compound **6** with the EGFR binding site.

### 2.6. Molecular Docking Study into the Tubulin Binding Site

**Figure 7:**
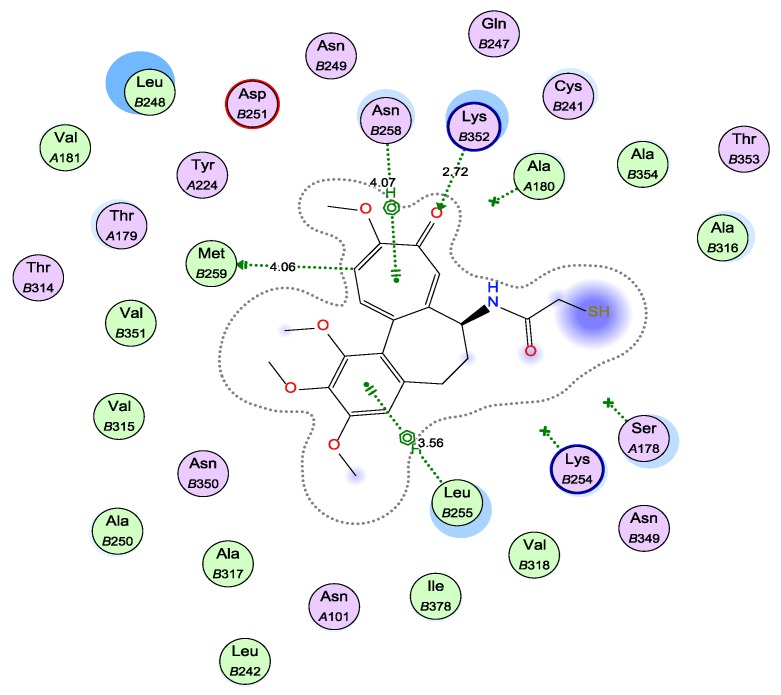
2D interaction of colchicine with tubulin binding site.

**Figure 8:**
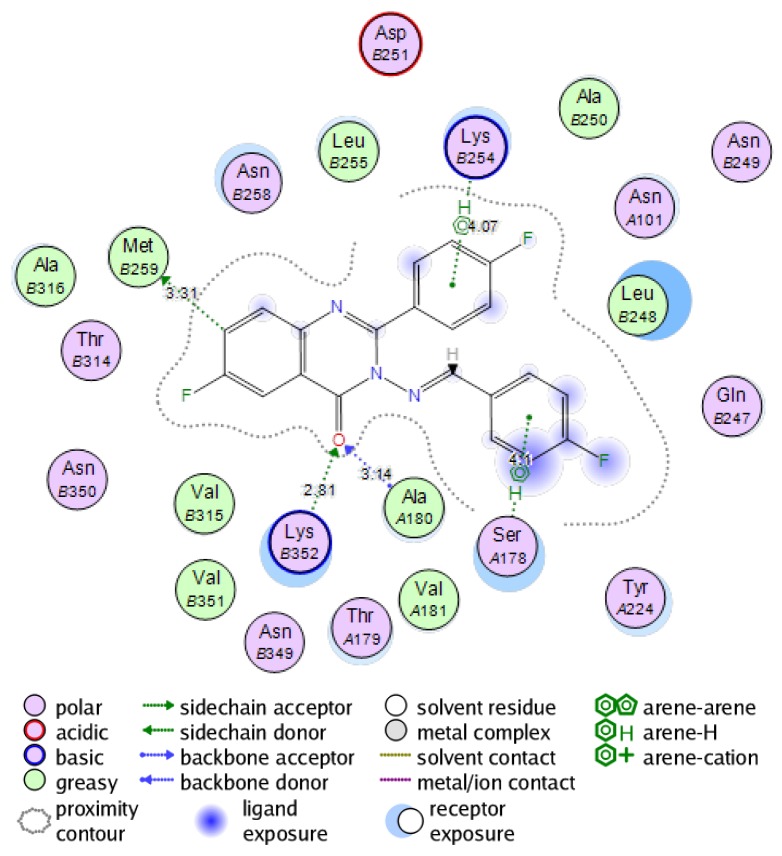
2D interactions of compound **10d** with tubulin binding site.

Compound **10d** was docked inside the binding site of colchicine in the crystalline structure of tubulin (PDB; 1SAO) using the AutoDock software to compare binding mode of this compound with that of colchicine. The co-crystallized colchicine revealed H bonding interaction between (C=O) and amino acid residue *βLys352* (distance 2.72 Å), aromatic hydrophobic interactions between the six-membered aromatic ring of colchicine and *βLeu255* (distance 3.56 Å) and aromatic hydrophobic interaction for the seven-membered aromatic ring of colchicine with both of *βMet259* (distance 4.06 Å) and *βAsn258* (distance 4.07 Å). When compound **10d** docked into this binding site, it occupied the same manner through formation of hydrogen bonding with the amino acid residue *βLys352* (distance 2.81 Å) in addition to hydrogen bonding with *αAla180* (distance 3.14 Å). There were also aromatic hydrophobic interactions between two aromatic rings and *βLys254* (distance 4.07 Å), *αSer178* (distance 4.1 Å) and *βMet259* (distance 3.31 Å). Binding energy score was −16.89 Kcal/mol for colchicine and −15.12 Kcal/mol for **10d**. The binding mode of this compound reflected strong activity as a tubulin polymerization inhibitor. Figure 7 and Figure 8 show 2D interactions of colchicine and compound **10d** with the tubulin binding site.

## 3. Materials and Methods

### 3.1. Chemistry

Chemicals were obtained from Sigma-Aldrich (St. Louis, MO, USA). All solvents were prepared according to standard methods. Aluminum sheets (Type 60 GF256, Merck, Kenilworth, NJ, USA) of pre-coated silica gel were used for TLC. Spots were identified by exposure to UV-lamp at λ 254 nm. Melting points were measured by Mel-Temp Sigma-Aldrich (St. Louis, MO, USA). and they are uncorrected. IR spectra were measured by using KBr discs and Perkin Elmer spectrophotometer (PerkinElmer, Melville, NY, USA). ^1^HNMR and ^13^CNMR spectra were measured by Bruker FT-NMR/400 (400 MHz) using DMSO-*d*_6_ as solvents and TMS as internal standard. Mass spectra were measured by Shimadzu (Kyoto, Japan) GC–MS/QP (70 eV). Analyses of C, H, N were measured by Varia elemental analyzer III (Varia, Hanau, Germany). Elemental analysis values were within ±0.4% of the theoretical values. All spectral analyses were done at micro-analytical center, Cairo, Egypt.

### 3.2. Synthesis of 6-Fluoro 2-(4-fluorophenyl)-benzo[d] [1,3] oxazine-4-one (***3***)

2-amino-5-fluorobenzoic acid **1** (0.1 mole) was dissolved in dry pyridine (30 mL), then it was added slowly with continuous stirring to a solution of 4-fluorobenzoyl chloride **2** (0.15 mole) in dry pyridine (30 mL). After complete addition, the mixture was subjected to strong stirring for 1 h. Then, solution of sodium bicarbonate (10%) was added slowly until the effervescence was stopped. The obtained solid was filtered off and washed with cold water repeatedly till there was no smell of pyridine or unreacted 4-flurobenzoyl chloride. The solid was dried and recrystallized from ethanol to afford pure sample of 2-amino-5-fluorobenzoic acid as white crystals.

Yield 81%; mp: 163–165 °C; IR (KBr, νmax, cm^−1^): 3058 (C–H), 1748 (C=O), 1512 (C=N), 1482 (C=C), 1365 (C–N). ^1^HNMR (DMSO-*d*_6_): δ 7.41–8.25 (m, 7H, Ar-H). ^13^C NMR (DMSO-*d*_6_): δ 124.5, 118.7, 124.5, 125.8, 12.8, 132.5, 136.1, 138.2, 140.5, 148.6, 150.2, 154.3, 158.4, 162.8. Anal. Calcd. For C_14_H_7_F_2_NO_2_ (259.04): C, 64.87; H, 2.72; N, 5.40. Found C, 64.72; H, 2.91; N, 5.67. MS (ESI) *m*/*z* 260.04 [M + 1].

### 3.3. Synthesis of Isomers of 2-(4-Fluorobenzamido)-5-fluorobenzohydrazide (***4***) and 3-Ami.no-6-fluoro-2-(4-fluorophenyl)quinazolin-4(3H)-one (***5***)

A mixture of 6-fluoro 2-(4-fluorophenyl)-benzo[d] [1,3] oxazine-4-one (**3**) (0.01 mol) and hydrazine hydrate (0.015 mol) in dry pyridine (30 mL) was heated under reflux for 4 h. Then, the solid was separated and filtered, washed with water, dried and crystallized from ethanol. Isomers were separated by column chromatography using benzene:acetone in 7:3 ratio.

#### 3.3.1. 2-(4-Fluorobenzamido)-5-fluorobenzohydrazide (**4**)

Yield 30%; 230–232 °C; IR (KBr, νmax, cm^−1^): 3218 (s), 1570 (b) (NH), 3060 (C–H), 1668 (C=O), 1480 (C=C). ^1^HNMR (DMSO-*d*_6_): δ 6.41 (s, 2H, NH2), 7.21–8.54 (m, 7H, Ar-H), 9.32 (s, 1H, –CONH–), 10.21 (s, 1H, –NHCO–). ^13^C NMR (DMSO-*d*_6_): δ 117.8, 119.8, 120.7, 124.5, 129.6, 130.8, 132.7, 136.5, 140.4, 142.7, 145.8, 162.2, 164.7, 1695. Anal. Calcd. For C_14_H_11_F_2_N_3_O_2_ (291.08): C, 57.73; H, 3.81; N, 14.43. Found C, 57.81; H, 4.01; N, 14.62. MS (ESI) *m*/*z* 292.08 [M + 1].

#### 3.3.2. Synthesis of 3-Amino-6-fluoro-2-(4-fluorophenyl)quinazolin-4(3*H*)-one (**5**)

6-fluoro 2-(4-fluorophenyl)-benzo[d] [1,3] oxazine-4-one (**3**) (0.01 mol) and hydrazine hydrate (0.015 mol) were fused together at 250 °C in an oil bath for 0.5 h. The mixture was left to cool then methanol was added to this mixture. The separated solid was collected, filtered, dried, and recrystallized from ethanol. 

Yield 40%; 235–237 °C; IR (KBr, νmax, cm^−1^): 3265 (s), 1580 (b) (NH_2_), 3072 (C–H), 1669 (C=O), 1564 (C=N), 1487 (C=C), 1378 (C–N). ^1^HNMR (DMSO-*d*_6_): δ 5.37 (s, 2H, NH2), 7.24–8.38 (m, 7H, Ar-H). ^13^C NMR (DMSO-*d*_6_): δ 119.7, 122.8, 124.9, 128.1, 130.9, 133.7, 134.6, 138.5, 144.7, 148.7, 149.8, 165.6, 167.7, 169.2. Anal. Calcd. For C_14_H_9_F_2_N_3_O (273.07): C, 61.54; H, 3.32; N, 15.38. Found C, 61.71; H, 3.55; N, 15.52. MS (ESI) *m*/*z* 274.07 [M + 1].

### 3.4. Synthesis of 3-(Substituted benzylideneamino)-6-fluoro-2-(4-fluorophenyl)quinazolin-4(3H)-ones (***6**–**8***) and (***10a**–**g***)

3-amino 6-fluoro-2-(4-fluorophenyl)quinazolin-4(3*H*)-one (**5**) and different aldehydes in equivalent molar quantities were mixed in glacial acetic acid and heated under reflux for 6 h. The mixture was cooled, poured carefully into crushed ice and left for a few minutes to separate the solid. The solid obtained was filtered off, washed repeatedly with water, dried and recrystallized from ethanol.

#### 3.4.1. 3-(3-Iminoindolin-2-one)-6-fluoro-2-(4-flurophenyl)quinazoline-4(3*H*)-one (**6**)

Yield 60%; mp 223–225 °C; IR (KBr, νmax, cm^−1^): 3056 (CH), 1646 (C=O), 1542 (C=N), 1496 (C=C), 1380 (C-N) ^1^HNMR (DMSO-*d*_6_): δ 6.65–8.37 (m, 11H, Ar-H), 10.27 (s, 1H, –NHCO–). ^13^C NMR (DMSO-*d*_6_): δ 117.6, 118.5, 121.9, 124.6, 125.8, 128.9, 129.5, 129.9, 131.4, 131.8, 132.6, 142.5, 146.1, 152.2, 153.6, 154.2, 162.3, 164.3,168.1, 168.9, 170.3, 174.6. Anal. Calcd. For C_22_H_12_F_2_N_4_O_2_ (402.09): C, 65.67; H, 3.01; N, 13.92. Found C, 65.81; H, 3.34; N, 13.82. MS (ESI) *m*/*z* 403.09 [M + 1].

#### 3.4.2. 3-((Furan-2-yl)methyleneamino)-6-fluoro-2-(4-fluorophenyl)quinazolin-4(3*H*)-one (**7**)

Yield 58%; mp 228–230 °C; IR (KBr, νmax, cm^−1^): 3055 (CH), 1661 (C=O), 1539 (C=N), 1487 (C=C), 1383 (C-N) ^1^HNMR (DMSO-*d*_6_): δ 6.95–8.17 (m, 10H, Ar-H), 8.65(s, 1H, N=CH). ^13^C NMR (DMSO-*d*_6_): δ 124.9, 125.4, 127.8, 129, 131.4, 132.8, 135.7, 140.9, 142.7, 146, 152.8, 154.9, 157.1, 161.7, 164.8, 167.7, 168.9, 170.3, 172.4. Anal. Calcd. For C_19_H_11_F_2_N_3_O_2_ (351.08): C, 64.96; H, 3.16; N, 11.96. Found C, 65.11; H, 3.05; N, 11.62. MS (ESI) *m*/*z* 352.08 [M + 1].

#### 3.4.3. 3-(3-Phenylallylideneamino)-6-fluoro-2-(4-fluorophenyl)quinazolin-4(3*H*)-one (**8**)

Yield 65%; mp 220–222 °C; IR (KBr, νmax, cm^−1^): 3050 (CH), 1652 (C=O), 1540 (C=N), 1489 (C=C), 1385 (C-N) ^1^HNMR (DMSO-*d*_6_): δ 6.35–9.57 (group of signals, 12H, Ar-H, olefinic CH=CH and N=CH). ^13^C NMR (DMSO-*d*_6_): δ 116.1, 118.2, 120.9, 124.4, 125.2, 127.3,129.2,129.9, 131.2, 132.7, 135.6, 140.4,143.4, 146.2, 150.2,153.9, 156.1, 160.3, 164.7,166.7, 168.6, 172.3, 174.5. Anal. Calcd. For C_23_H_15_F_2_N_3_O (387.12): C, 71.31; H, 3.90; N, 10.85. Found C, 71.01; H, 3.75; N, 10.92. MS (ESI) *m*/*z* 388.12 [M + 1].

#### 3.4.4. 3-(Benzylideneamino)-6-fluoro-2-(4-fluorophenyl)quinazolin-4(3*H*)-one (**10a**)

Yield 64%; mp 218–220 °C; IR (KBr, νmax, cm^−1^): 3062 (CH), 1665 (C=O), 1543 (C=N), 1482 (C=C), 1388 (C-N) ^1^HNMR (DMSO-*d*_6_): δ 6.75–8.07 (m, 12H, Ar-H), 8.72(s, 1H, N=CH). ^13^C NMR (DMSO-*d*_6_): δ 124.2, 125.2, 127.6, 129.1, 130.9, 132.6, 135.6, 138.7, 140.9, 142.7, 145.6, 146.2, 152.6, 154.7, 158.9, 162.8, 165.1, 166.9, 168.7, 170.2, 172.6. Anal. Calcd. For C_21_H_13_F_2_N_3_O_2_ (361.10): C, 69.80; H, 3.63; N, 11.63. Found C, 69.61; H, 3.45; N, 11.42. MS (ESI) *m*/*z* 362.10 [M + 1].

#### 3.4.5. 3-(4-Methylbenzylideneamino)-6-fluoro-2-(4-fluorophenyl)quinazoline-4(3*H*)-one (**10b**)

Yield 60%; mp 231–233 °C; IR (KBr, νmax, cm^−1^): 3058 (CH), 1662 (C=O), 1550 (C=N), 1486 (C=C), 1383 (C-N). ^1^HNMR (DMSO-*d*_6_): δ 2.48 (s, 3H, CH_3_), 7.05–8.13 (m, 11H, Ar-H), 8.81 (s, 1H, N=CH). ^13^C NMR (DMSO-*d*_6_): δ 22.29, 114.7, 118.2, 122.6, 128.1, 130.7, 132.2, 136.9, 138.2, 140.8, 142.2, 146.9, 148.2, 150.8, 156.5, 158.3, 160.8, 165.4, 166.3, 168.8, 170.7, 172.5. Anal. Calcd. For C_22_H_15_F_2_N_3_O (375.12): C, 70.39; H, 4.03; N, 11.19. Found C, 70.08; H, 3.95; N, 11.32. MS (ESI) *m*/*z* 376.12 [M + 1].

#### 3.4.6. 3-(4-Methoxybenzylideneamino)-6-fluoro-2-(4-fluorophenyl)quinazolin-4(3*H*)-one (**10c**)

Yield 62%; mp 228–230 °C; IR (KBr, νmax, cm^−1^): 3048 (CH), 1659 (C=O), 1553 (C=N), 1479 (C=C), 1380 (C-N) ^1^HNMR (DMSO-*d*_6_): δ 3.69 (s, 3H, OCH_3_), 6.95–8.01 (m, 11H, Ar-H), 8.76 (s, 1H, N=CH). ^13^C NMR (DMSO-*d*_6_): δ 52.24, 115.2, 118.7, 122.6, 124.7, 128.7, 130.5, 132.6, 134.9, 138.6, 140.8, 142.6, 146.3, 148.7, 156.7, 158.1, 160.9, 164.4, 166.1, 168.6, 171.9, 172.8. Anal. Calcd. For C_22_H_15_F_2_N_3_O_2_ (391.37): C, 67.52; H, 3.86; N, 10.74. Found C, 67.37; H, 3.98; N, 11.01. MS (ESI) *m*/*z* 392.37 [M + 1].

#### 3.4.7. 3-(4-Fluorobenzylideneamino)-6-fluoro-2-(4-fluorophenyl)quinazolin-4(3*H*)-one (**10d**)

Yield 58%; mp 232–234 °C; IR (KBr, νmax, cm^−1^): 3051 (CH), 1655 (C=O), 1551 (C=N), 1482 (C=C), 1386 (C-N) ^1^HNMR (DMSO-*d*_6_): δ 6.89–8.21 (m, 11H, Ar-H), 8.83 (s, 1H, N=CH). ^13^C NMR (DMSO-*d*_6_): δ 116.2, 118.9, 122.5, 125.1, 128.7, 130.8, 132.8, 136.9, 140.7, 142.4, 146.8, 148.9, 152.6, 154.8, 156.3, 160.1, 164.7, 167.1, 169.4, 171.7, 172.9. Anal. Calcd. For C_21_H_12_F_3_N_3_O (379.09): C, 66.49; H, 3.19; N, 11.08. Found C, 66.27; H, 3.48; N, 10.89. MS (ESI) *m*/*z* 380.09 [M + 1].

#### 3.4.8. 3-(4-Nitrobenzylideneamino)-6-fluoro-2-(4-fluorophenyl)quinazolin-4(3*H*)-one (**10e**)

Yield 60%; mp 235–237 °C; IR (KBr, νmax, cm^−1^): 3054 (CH), 1653 (C=O), 1560 (C=N), 1480 (C=C), 1381 (C-N) ^1^HNMR (DMSO-*d*_6_): δ 6.68–8.09 (m, 11H, Ar-H), 8.69 (s, 1H, N=CH). ^13^C NMR (DMSO-*d*_6_): δ 115.1, 116.9, 118.6, 123.8, 126.1, 128.8, 130.9, 134.3, 136.6, 140.5, 142.8, 146.6, 148.7, 152.8, 157.3, 160.2, 164.6, 167.3, 169.2, 170.6, 172.8. Anal. Calcd. For C_21_H_12_F_2_N_4_O_3_ (406.09): C, 62.07; H, 2.98; N, 13.79. Found C, 62.37; H, 3.18; N, 13.92. MS (ESI) *m*/*z* 407.09 [M + 1].

#### 3.4.9. 3-(4-Chlorobenzylideneamino)-6-fluoro-2-(4-fluorophenyl)quinazolin-4(3*H*)-one (**10f**)

Yield 64%; mp 224–226 °C; IR (KBr, νmax, cm^−1^): 3050 (CH), 1649 (C=O), 1557 (C=N), 1478 (C=C), 1387 (C-N) ^1^HNMR (DMSO-*d*_6_): δ 6.91–8.29 (m, 11H, Ar-H), 8.71 (s, 1H, N=CH). ^13^C NMR (DMSO-*d*_6_): δ 116.5, 117.9, 119.2, 122.8, 124.2, 126.9, 131.7, 134.6, 136.9, 139.5, 142.7, 145.4, 148.9, 152.7, 158.1, 160.4, 163.9, 167.5, 169.3, 171.1, 172.8. Anal. Calcd. For C_21_H_12_ClF_2_N_3_O (395.06): C, 63.73; H, 3.06; N, 10.62. Found C, 63.87; H, 3.21; N, 10.41. MS (ESI) *m*/*z* 396.06 [M + 1].

#### 3.4.10. 3-(4-Hydroxybenzylideneamino)-6-fluoro-2-(4-fluorophenyl) quinazolin-4(3*H*)-one (**10g**)

Yield 68%; mp 229–231 °C; IR (KBr, νmax, cm^−1^): 3057 (CH), 1651 (C=O), 1564 (C=N), 1475 (C=C), 1395 (C-N) ^1^HNMR (DMSO-*d*_6_): δ 7.11–8.69 (m, 11H, Ar-H), 8.74 (s, 1H, N=CH), 11.51 (s, 1H, OH). ^13^C NMR (DMSO-*d*_6_): δ 114.1, 116.3, 118.2, 120.2, 122.9, 124.7, 126.8, 131.8, 133.8, 135.9, 138.5, 142.6, 146.1, 148.8, 153.8, 157.7, 160.6, 163.7, 166.5, 168.6, 170.1. Anal. Calcd. For C_21_H_13_F_2_N_3_O_2_ (377.09): C, 66.84; H, 3.47; N, 11.14. Found C, 66.57; H, 3.61; N, 11.24. MS (ESI) *m*/*z* 378.09 [M + 1].

### 3.5. In Vitro Cytotoxic Screening

The in vitro cytotoxic screening of target compounds was performed against two breast cancer cell lines MCF-7 and (MDA-MB-231). Cell lines were obtained from American Type Culture Collection, cells were cultured using DMEM (Invitrogen Life Technologies, Waltham, MA, USA) supplemented with 10% FBS (Hyclone, Logan, UT, USA), 10 µg/mL of insulin (Sigma, St. Louis, MO, USA), and 1% penicillin-streptomycin. The cells were sub-cultured into a 96-well plate with 1104 cells per well in medium, at 37 °C, 5% CO_2_ and 95% air atmosphere, before being treated with or without various concentrations of test compounds, each in triplicate for 24 h. At the end of the incubation, the cells were harvested and washed with PBS. 20 µL of MTT was added to each well and incubated for 2 h before 200 µL DMSO was added. The absorbance was measured on an ELISA reader (Multiskan EX, Lab systems, Waltham, MA, USA) at wavelength of 570 nm [17,18].

### 3.6. EGFR Inhibition Assay

EGFR kinase activity was measured by HT Scan EGFR kinase assay kits (Cell Signaling Technology, Danvers, MA, USA). The experiments were performed following the manufacturer’s directions. In conclusion, Synthetic substrate, 10 μg/mL inhibitors and the glutathione s-transferase (GST)-EGFR protein were incubated in the presence of 400 μM ATP. Strapavidin-coated 96-well plates were used to take phosphorylated substrate. Anti-phosphotyrosine and europium-labeled secondary antibodies (DELFIA, Perkin-Elmer, Akron, OH, USA) were used to monitor the level of phosphorylation. At the end of the assay, the enrichment solution was added, and enzyme activity was assessed in the Wallac Victor II 1420 micro-plate reader (Abcam, Milton, Cambridge, UK) at 615 nM [19].

### 3.7. Tubulin Polymerization Inhibition Assay

The microtubule polymerization assay was carried out in 96-well plates at 37 °C, with 1 mg/mL bovine microtubule-associated protein (MAP)-rich tubulin (Cytoskeleton) and the indicated test compound in PEM buffer [80 mM PIPES (pH 6.8), 1 mM EGTA, and 1 mM MgCl_2_] containing 1 mM GTP. To measure the enhancement of polymerization, the microtubule polymerization assay was carried out in 96-well plates at 25 °C, with 1 mg/mL bovine MAP-rich tubulin and the indicated test compound in PEM buffer. Tubulin polymerization was monitored by changes in absorbance at 340 nm [19].

### 3.8. Statistical Analysis

All assays were performed in triplicate. The results were expressed as mean ± SD (standard deviation) using Student’s *t* test.

### 3.9. Molecular Docking

Molecular docking simulation was carried out using the program AutoDock 4.0.1.34 (version 4.0, Molecular graphics laboratories, La Jolla, CA, USA) with the graphical user interface AutoDock tools (ADT) [20,21,22,23]. The active compounds were docked into (3D) complex of the two biological targets: crystal EGFR (PDB code: 1M17) complexes with erlotinib at 2.6 Å resolution and crystal structure tubulin (PDB: 1SAO) complexes with colchicine at 3.5 Å resolution [19,21]. The ligand and solvent molecules were removed from the crystal structure to obtain the docking grid and the active site was defined using AutoGrid [22]. The grid box was centered on the center of the ligand from the corresponding crystal structure complexes. The Lamarckian genetic algorithm issued for docking with the following settings: a maximum number of 2,500,000 energy evaluations, an initial population of 50 randomly placed individuals, a maximum number of 37,000 generations, a mutation rate of 0.02, across over rate of 0.80, and an elitism value (number of top individuals that automatically survive) of 1. The ligand was fully optimized inside the binding site during the docking simulations, the conformation with the lowest predicted binding free energy of the most occurring binding modes in erlotinib active pocket was selected and hydrogen atoms were added to the structure using the Molecular Operating Environment (MOE 2012) [23,24,25]. Selected active compounds were docked into the active site of the two targets to predict compound binding modes. 

## 4. Conclusions

Some new derivatives of substituted fluoroquinazolinone (**6**–**8**) and (**10a**–**g**) were designed, synthesized and biologically screened as cytotoxic agents against two cancer cell lines MCF-7 and MDA-MBA-231. All these derivatives showed significant cytotoxic activity with variable IC_50_ values ranging from 0.28 ± 0.02 µM to 36.57 ± 1.81 µM against the two cell lines. Some compounds—**6**, **7**, **10a**, **10c**, **10d**, **10e** and **10f**—had better cytotoxic activity on the two cell lines than the reference gefitinib. EGFR assay of the highest active compounds displayed excellent activity comparing to the reference gefitinib. Tubulin polymerization inhibition assay showed good results for these derivatives as cytotoxic agents having an ability to stop mitotic division by inhibition of tubulin polymerization. Molecular docking of the highly active compounds explained the mode of binding of these derivatives. This mode has the same manner as the reference drug in addition to extra hydrogen bonds that formed stronger ligand-receptor interactions. In conclusion, the highest active compounds could be subjected to future optimization and investigation to be effective antitumor drugs.

## Figures and Tables

**Table 1:** IC_50_ values for cytotoxic screening of title compounds against two cell lines (MCF-7) and (MDA-MBA-231).

Compound	IC_50_ µM		3rd Position Substitution
	MCF-7	MDA-MBA-231	
**6**	0.35 ± 0.01	1.38 ± 0.14	3-iminoindolin-2-one
**7**	1.06 ± 0.03	0.94 ± 0.07	(furan-2-yl)methylene amine
**8**	36.57 ± 1.81	21.64 ± 1.4	Phenylallylidene amine
**10a**	0.95 ± 0.01	2.48 ± 0.17	Substituted benzylidene amine
**10b**	5.07 ± 0.33	3.09 ± 0.08	
**10c**	10.43 ± 1.14	1.09 ± 0.01	
**10d**	0.89 ± 0.02	0.38 ± 0.01	
**10e**	2.61 ± 0.14	0.28 ± 0.02	
**10f**	0.71 ± 0.01	3.76 ± 0.22	
**10g**	1.32 ± 0.08	2.54 ± 0.23	
Gefitinib	0.9 ± 0.02	1.30 ± 0.04	

**Table 2:** Selectivity indices for the title compounds arranged according to type of selectivity. S1 = IC_50_ (MCF-7)/IC_50_ (MDA-MBA-231) while S2 = IC_50_ (MDA-MBA-231)/IC_50_ (MCF-7). When S1 > S2, the compound is more selective to MDA-MBA-231, and when S2 > S1, the compound is more selective to MCF-7. Values are expressed as the mean ± SD of at least three independent experiments.

Compound	Selectivity Indices		Cell Line
	S1	S2	
**10c**	9.57 ± 0.11	0.10 ± 0.13	MDA-MBA-231 selective
**10e**	9.32 ± 0.08	0.11 ± 0.17	
**10d**	2.34 ± 0.05	0.43 ± 0.09	
**8**	1.69 ± 0.15	0.59 ± 0.02	
**10b**	1.64 ± 0.21	0.61 ± 0.06	
**7**	1.13 ± 0.09	0.89 ± 0.18	
Gefitinib	0.75 ± 0.18	1.34 ± 0.19	MCF-7 selective
**10g**	0.52 ± 0.07	1.92 ± 0.05	
**10a**	0.39 ± 0.01	2.61 ± 0.09	
**6**	0.25 ± 0.06	3.94 ± 0.26	
**10f**	9.57 ± 0.22	5.29 ± 0.31	

**Table 3:** IC_50_ values of EGFR assay for the most active compounds—**6** and **10e**—and the reference gefitinib. Values are expressed as the mean ± SD of at least three independent experiments.

Compound	IC_50_ (nM)	
	MCF-7	MDA-MBA-231
**6**	75.2 ± 0.08	-
**10e**	-	170.08 ± 0.02
Gefitinib	78.04 ± 0.11	299 ± 0.12
